# Health literacy among adolescents: summary of some key findings from ten European countries

**DOI:** 10.1093/eurpub/ckac129.321

**Published:** 2022-10-25

**Authors:** L Paakkari, H Lahti, M Kulmala, O Paakkari, N Lyyra

**Affiliations:** Faculty of Sport and Health Sciences, University of Jyväskylä, Jyväskylä, Finland

## Abstract

**Introduction:**

In research on disparities, the concept of health literacy (HL) as a set of competencies to promote and sustain health may help in understanding the disparities better and in addressing avoidable and unfair health disparities. The presentation will present some key findings on adolescents’ HL levels in ten European countries, and how HL mediates and moderates between various background factors and health outcomes.

**Methods:**

Data consisted of cross-sectional data from Health Behaviour in School-aged Children (HBSC) study from year 2017/18 of ten European countries (Austria, Belgium (Fl), Czechia, England, Estonia, Finland, Germany, Macedonia, Poland, and Slovakia). Data (n = 14,590 - 22,291) of 13- and 15-year-old pupils were used. Indicators include background variables (e.g. age, gender), Health literacy, Health indicators (e.g. self-rated health (SRH) and problematic social media use (PSMU)). Analysis include (1) Mediator analysis (with Mplus): pearson correlation coefficients, path models (Mplus 7.3 and Maximum Likelihood estimator) and (2) random effects models and moderator analyses (with R-sofware).

**Findings:**

HL is an independent factor explaining disparities in health (e.g. SRH), and a mediator as well as a moderator between health outcomes and background factors. Based on the national analyses HL had significant main effects in every country, but group level differences emerged only in some countries. For instance, in Finland and Belgium, among girls HL lowered the likelihood to problematic social media use, but not among boys.

**Discussion:**

HL is of use in understanding and tackling health disparities among adolescents. Results confirm the need to adopt the principles of proportionate universalism when promoting HL among adolescents to avoid widening the disparities within population groups. Also, country-specific health literacy interventions are needed to secure equity in opportunities of different population groups to benefit from the HL interventions.

